# Prevalence of Anxiety and Depression in Patients With Multiple Sclerosis in Saudi Arabia: A Cross-Sectional Study

**DOI:** 10.7759/cureus.20792

**Published:** 2021-12-29

**Authors:** Rahaif H Aljishi, Rahaf J Almatrafi, Zainab A Alzayer, Bayan A Alkhamis, Esraa E Yaseen, Amal M Alkhotani

**Affiliations:** 1 Department of Medicine, Faculty of Medicine, Hai'l University, Hai'l, SAU; 2 Department of Medicine, Faculty of Medicine, Umm Al-Qura University, Makkah, SAU; 3 Department of Medicine, Faculty of Medicine, Ibn Sina National College for Medical Studies, Jeddah, SAU

**Keywords:** saudi arabia, severity, anxiety, depression, multiple sclerosis

## Abstract

Introduction

Multiple sclerosis (MS) is a chronic disease of progressive demyelination in the central nervous system and carries a significant risk for depression and other psychological difficulties associated with low quality of life. There is a paucity of data on the prevalence of anxiety and depression in Saudi Arabia among patients with MS. We conducted a cross-sectional study to determine the prevalence of anxiety and depression in Saudi Arabia among patients with MS by age, disease severity, compliance to medication, and social support.

Methods

This cross-sectional study measured the prevalence of anxiety and depression in 184 adult patients with MS. The patients were selected through a random sampling method from a pool of MS societies in Saudi Arabia. The participants completed self-administered questionnaires that included demographic variables. The participants also completed the Patient Health Questionnaire-9 (PHQ-9) and the General Anxiety Disorder-7 (GAD-7) questionnaire.

Results

Depression was detected among 139 (75.5%) patients with MS, with most participants having mild depression (31%). More women (83.1%) experienced depression than men (62.1%; p = 0.002). Anxiety disorder was present in 123 (66.8%) patients with MS, and most had mild anxiety (n = 56; 30.4%).

Conclusion

We found a very high rate of depression and anxiety among patients with MS in Saudi Arabia. Our results highlight the need for periodic screening and examination of patients with MS by psychiatrists to facilitate the early detection and treatment of these comorbidities, potentially improving patient quality of life and health outcomes.

## Introduction

Multiple sclerosis (MS) is a chronic disease of progressive demyelination in the central nervous system that shrinks the neuronal sheath and plaque formation in different parts of the brain [1,2]. MS was considered one of the most common neurological disorders and is typically diagnosed in early adulthood [3]. MS occurs in 57-78 per 100,000 people and affects an estimated 2.5 million people globally [2].

The etiology of MS is still unknown, but several factors most likely contribute to the disease, including genetics (e.g., the presence of the HLA-DRB1 allele), environmental risk factors (e.g., Epstein-Barr virus and low levels of vitamin D), or behavioral factors (e.g., cigarette smoking) [4,5]. The most common symptoms are impaired vision, double vision, limb weakness, gait disability, and bowel/bladder symptoms [6]. Psychological difficulties are a significant concern for patients with MS; most suffer from anxiety, depression, and stress [2]. The lifetime risk of major depression in people with MS is estimated at 50% compared to the 10%-15% risk of major depression in the general population. Thus, depression plays a critical role in determining the quality of life in patients with MS. When depression is concurrent with anxiety, patients with MS are at an elevated risk of suicide [7].

While still lower than the western and neighboring countries, the prevalence of MS in Saudi Arabia is 40.40 per 100,000 residents, placing the Kingdom above the low-risk zone per the Kurtzke classification [8]. The prevalence of anxiety and depression in Saudi Arabia among patients with MS has not been well studied [3,7,9]. Therefore, we conducted this study to determine the prevalence of anxiety and depression in Saudi Arabia among patients with MS according to age, disease severity, compliance to medication, and social support using a large sample size.

## Materials and methods

We conducted a cross-sectional study of 184 adult patients affected by MS (66 men and 118 women), selected through a random sampling method from the MS societies in Saudi Arabia. The participants completed self-administered questionnaires from August 2021 to October 2021. We included patients in Saudi Arabia diagnosed with MS who were older than age 18, and any patients who were not Saudi or did not complete the questionnaire in full were excluded from the study population.

We calculated our sample size using the standard single proportion formula at a confidence level of 95%, precision of 5%, and significance level of 0.05, adding 10% to the original number to compensate for possible losses. Our sample size was limited to 184 due to our criteria for this research, especially since many patients with MS were non-Saudi while our research focused on Saudi individuals only. Data extraction and cleaning of those who did not meet the criteria also resulted in a further reduction in numbers. Before data collection, we obtained Institutional Review Board approval from Umm Al-Qura University Research Ethics Committee in Makkah City, explained the study objectives to the patients, and obtained their voluntary consent before enrolling them in the study.

Questionnaires

We asked patients to complete an online questionnaire that included demographic variables such as age, sex, marital status, education, and job status. We also collected specific information regarding MS clinical features (e.g., type, onset, duration, pharmacotherapy, and health status compared to the previous year) and other variables, such as comorbidities and physical health.

The patients also completed the Patient Health Questionnaire-9 (PHQ-9) and the General Anxiety Disorder-7 (GAD-7) questionnaire. Both questionnaires were translated into Arabic. The PHQ-9 is a short, self-administered scale based on the nine Diagnostic and Statistical Manual of Mental Disorders-IV criteria for diagnosing depression, with a suggested cutoff score of 10 [10]. The PHQ-9 has a sensitivity of 88% and specificity of 88% for severe depression, making it a suitable tool for screening for depression in patients with MS [11]. The GAD-7 is a short, self-administered scale with a cutoff point of 10 that has a sensitivity of 89% and specificity of 82% for diagnosing generalized anxiety disorder [12]. The GAD-7 has improved reliability and internal validity in previous studies involving patients with MS [13].

Data analysis

We used IBM SPSS Statistics for Windows version 22.0. (IBM Corp., Armonk, NY, USA) to analyze the collected data. Two-tailed tests were used for all statistical analyses. A p-value of less than 0.05 was considered statistically significant. The frequency and percent distribution of descriptive analysis was done for all variables, including patients' data, MS medical health conditions, and social support. As for the patient health questionnaire, the discrete scores for different items were summed to achieve an overall score. Based on the questionnaire-reported cutoff points, the overall score was categorized into no/minimal depression, mild depression, moderate depression, moderately severe depression, and severe depression [10,14]. Also, the GAD-7 discrete item scores were summed to have an overall score categorized into no/minimal anxiety, mild anxiety, moderate anxiety, and severe anxiety, in reference to the tool-reported cutoff value [13,15]. Cross-tabulation was used to assess the distribution of depression and anxiety of patients with MS by their personal and other related data. The significance of relations in cross-tabulation was tested using the Pearson chi-square test and exact probability test for small frequency distributions.

## Results

A total of 184 patients with MS fulfilled the inclusion criteria and participated in the study. The patients ranged from 18 to 59 years old (mean age: 34.9 ± 11.7 years). Most participants (n = 118; 64%) were women; 66 were men (35.9%). Most participants were married, had a university level of education or above, and were unemployed. All patient demographic information and self-perceived mental health data are presented in Table 1. Regarding self-ranking of mental health, 105 (57.1%) patients thought they were depressed, and 117 (63.6%) thought they had an anxiety disorder at the time of survey completion.

**Table 1:** Demographic data of patients diagnosed with multiple sclerosis in Saudi Arabia

Demographic data	N	%
Age in years		
18–25	42	22.8%
26–34	54	29.3%
35–43	55	29.9%
44+	33	17.9%
Gender		
Male	66	35.9%
Female	118	64.1%
Educational level		
Secondary/below	57	31%
University/above	127	69%
Work		
Not working	106	57.6%
Full-time work	59	32.1%
Part-time work	9	4.9%
Free works	10	5.4%
Marital status		
Single	70	38%
Married	98	53.3%
Divorced/widow	16	8.7%
Do you think you depressed now?		
Yes	105	57.1%
No	79	42.9%
Do you think you suffer from an anxiety disorder?		
Yes	117	63.6%
No	67	36.4%

Table 2 presents MS-related data among the study participants. While the majority of patients (73.4%) did not know their type of MS, among those who knew their MS type, the most common was relapsing-remitting MS (RRMS) (15.2%), followed by progressive-relapsing MS (PRMS) (3.3%), secondary progressive MS (SPMS) (3.3%), primary progressive MS (PPMS) (2.7%), and fulminant type MS (FTMS) (2.2%). Most patients (n = 132; 71.7%) had been diagnosed over two years before participating in the study, and most (n = 159; 86.4%) had been receiving MS treatment.

**Table 2:** Multiple sclerosis-related data among the study patients in Saudi Arabia MS: multiple sclerosis

Clinical data	N	%
Type of multiple sclerosis		
Remitting-relapsing MS	28	15.2%
Progressive-relapsing MS	6	3.3%
Secondary progressive MS	6	3.3%
Primary progressive MS	5	2.7%
Fulminant type MS	4	2.2%
Don't know	135	73.4%
Duration of MS		
<2 years	52	28.3%
>2 years	132	71.7%
Treatment situation		
Not on treatment	25	13.6%
Still on treatment	159	86.4%
How would you rate your health in general?		
Poor	21	11.4%
Good	88	47.8%
Very good	48	26.1%
Excellent	27	14.7%
How would you rate your health now compared to last year?		
Much better than last year	29	15.8%
Somewhat better than last year	40	21.7%
No change	62	33.7%
Somewhat worse	30	16.3%
Much worse	23	12.5%

Table 3 presents social support for patients with MS. Only 65 (35.3%) patients with MS reported social support. Among them, most reported that they had a person who listened to them (89.2%), gave them love and attention (87.7%), they trusted and were able to talk to comfortably (86.2%), prepared them a meal when needed (84.6%), gave advice (80%), and take them to doctor's appointments (83.1%).

**Table 3:** Social support for patients with multiple sclerosis in Saudi Arabia

Social support	N	%
Had social support		
Yes	65	35.3%
No	119	64.7%
Do you have someone who(m): (n = 65)		
Helps you when you stay in bed too long?	47	72.3%
Take you to the doctor?	54	83.1%
Prepares your meals if you cannot make them?	55	84.6%
Helps you in your daily affairs if you are ill?	52	80%
Hears you when you need to talk?	49	75.4%
Gives you good advice in difficult times?	52	80%
Gives you love and attention?	57	87.7%
You can listen to?	58	89.2%
Gives you information that helps you understand a topic?	54	83.1%
You trust and trusts you to talk about themselves and their problems with you?	56	86.2%
Hugs you?	46	70.8%
You can relax with?	51	78.5%
Gives you advice that you value?	55	84.6%
Helps you clear your mind?	52	80%
You share your deepest fears and personal problems?	45	69.2%
Guides you on how to solve some of your problems, someone who shares the things that make you happy?	53	81.5%
Understands you and your problems?	51	78.5%

The PHQ-9 results are presented in Table 4. The mean PHQ-9 score was 10.5 (range: 0-27). Most patients (83.2%) reported feeling tired; 70.1% felt down, depressed, or hopeless; 67.9% had trouble falling or staying asleep, or sleeping too much; 65.2% had little interest or pleasure in doing things; 64.7% had poor appetite or overeating; and 58.7% had trouble concentrating on activities such as reading the newspaper or watching television. Only 32.1% thought they would be better off dead or of hurting themselves in some way.

**Table 4:** Patient Health Questionnaire-9 item distribution among patients with multiple sclerosis in Saudi Arabia PHQ-9: Patient Health Questionnaire-9; SD: standard deviation

PHQ-9 items	Not at all		Several days		More than half the days		Nearly every day	
	N	%	N	%	N	%	N	%
Little interest or pleasure in doing things	64	34.8%	62	33.7%	22	12%	36	19.6%
Feeling down, depressed, or hopeless	55	29.9%	59	32.1%	33	17.9%	37	20.1%
Trouble falling or staying asleep, or sleeping too much	59	32.1%	44	23.9%	23	12.5%	58	31.5%
Feeling tired or having little energy	31	16.8%	54	29.3%	32	17.4%	67	36.4%
Poor appetite or overeating	65	35.3%	57	31%	26	14.1%	36	19.6%
Feeling bad about yourself – or that you are a failure or have let yourself or your family down	79	42.9%	47	25.5%	23	12.5%	35	19%
Trouble concentrating on things, such as reading the newspaper or watching television	76	41.3%	51	27.7%	19	10.3%	38	20.7%
Moving or speaking so slowly that other people could have noticed, or the opposite – being so fidgety or restless that you have been moving around a lot more than usual	102	55.4%	36	19.6%	12	6.5%	34	18.5%
Thoughts that you would be better off dead or of hurting yourself in some way	125	67.9%	21	11.4%	14	7.6%	24	13%
Overall score (range)	0–27							
Mean ± SD	10.5 ± 7.8							

**Figure 1:**
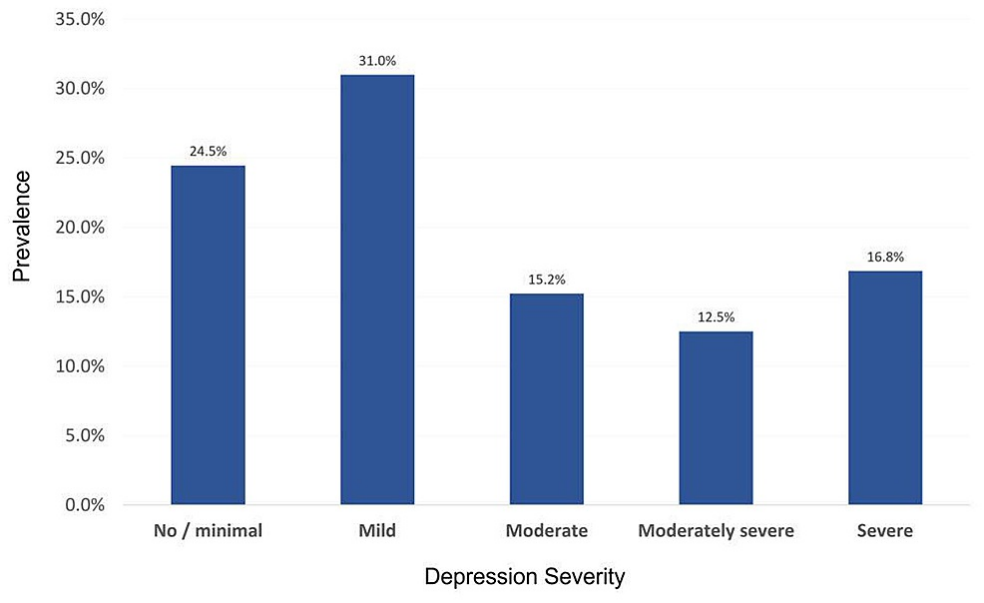
Depression severity Distribution of depression according to severity in patients with multiple sclerosis

Depression prevalence and severity data are presented in Figure 1. Depression was detected among 139 (75.5%) patients with MS, with most participants having mild depression (31%).

Table 5 presents the GAD-7 results among our study participants. The mean GAD-7 score for all patients was 8.5 of 21. Most participants (73.9%) reported they were easily annoyed or irritable; 67.9% had trouble relaxing; 65.2% felt nervous, anxious, or on edge; 64.7% were unable to stop or control worrying; 64.1% felt afraid as if something awful might happen; and 52.2% are so restless that it was challenging to sit still.

**Table 5:** General Anxiety Disorder item distribution among patients with multiple sclerosis in Saudi Arabia SD: standard deviation

GAD-7 items	Not at all		Several days		More than half the days		Nearly every day	
	N	%	N	%	N	%	N	%
Feeling nervous, anxious, or on edge	64	34.8%	61	33.2%	23	12.5%	36	19.6%
Not being able to stop or control worrying	65	35.3%	64	34.8%	27	14.7%	28	15.2%
Trouble relaxing	59	32.1%	55	29.9%	28	15.2%	42	22.8%
Being so restless that it's hard to sit still	88	47.8%	48	26.1%	21	11.4%	27	14.7%
Becoming easily annoyed or irritable	48	26.1%	64	34.8%	17	9.2%	55	29.9%
Feeling afraid as if something awful might happen	66	35.9%	49	26.6%	23	12.5%	46	25%
Overall score (range)	0–21							
Mean ± SD	8.5 ± 6.4							

**Figure 2:**
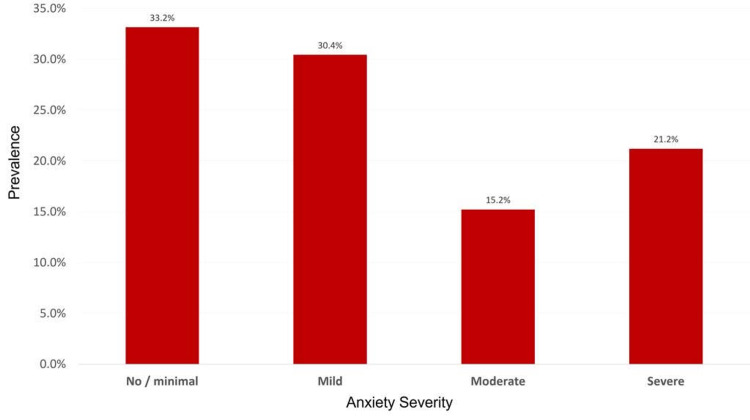
Anxiety severity Distribution of anxiety according to severity in patients with multiple sclerosis

Figure 2 presents the prevalence and severity of anxiety among the study participants. Anxiety disorder was present in 123 (66.8%) patients with MS, and most had mild anxiety (n = 56; 30.4%).

The distribution of depression and anxiety of patients with MS according to demographic data is presented in Table 6. A higher percentage of women (83.1%) had depression than men (62.1%; p = 0.002). There was a significant association between the type of MS and the prevalence of depression (0.004). There was a significant association between how patients rated their health and the prevalence of depression (p = 0.002). Depression was detected among 94.3% of patients who think they are depressed and 85.5% of those who think they had an anxiety disorder (p = 0.001). Considering anxiety disorder, 75.4% of women had anxiety compared with 51.5% of men (p = 0.001). Also, 80.7% of patients with a secondary level of education showed anxiety disorder compared with 60.6% of those with a higher educational level (p = 0.007). There was a significant relationship between the employment status of patients with MS and the prevalence of anxiety among them (p = 0.002). There was a significant association between marital status and the prevalence of anxiety among the patients with MS (p = 0.026). There was a significant association between the type of MS and the prevalence of anxiety (p = 0.001). There was a significant association between how patients rated their health and the prevalence of anxiety (p = 0.002). There was a significant association between how patients rated their health compared to the previous year and the prevalence of anxiety (55.2%; p = 0.028). Anxiety disorder was detected among 85.7% of patients who think they are depressed and 80.3% of those who think they had an anxiety disorder (p = 0.001 for both associations).

**Table 6:** Distribution of depression and anxiety of patients with MS by their demographic data MS: multiple sclerosis; PRMS: progressive-relapsing MS; RRMS: remitting-relapsing MS; PPMS: primary progressive MS; FTMS: fulminant type MS; SPMS: secondary progressive MS ^a^Statistically significant (p < 0.05) ^b^Exact probability test

Factors		Depressed		Anxiety	
		N	%	N	%
Age in years	18–25	35	83.3%	33	78.6%
	26–34	38	70.4%	32	59.3%
	35–43	43	78.2%	37	67.3%
	44+	23	69.7%	21	63.6%
p-value		0.395		0.244	
Gender	Male	41	62.1%	34	51.5%
	Female	98	83.1%	89	75.4%
p-value		0.002^a^		0.001^a^	
Educational level	Secondary/below	44	77.2%	46	80.7%
	University/above	95	74.8%	77	60.6%
p-value		0.727		0.007^a^	
Work	Not working	86	81.1%	80	75.5%
	Full-time work	40	67.8%	35	59.3%
	Part-time work	7	77.8%	6	66.7%
	Freelancer	6	60%	2	20%
p-value		0.169^b^	0.002^a,b^		
Marital status	Single	56	80%	53	75.7%
	Married	71	72.4%	57	58.2%
	Divorced/widow	12	75.0%	13	81.3%
p-value		0.532		0.026^a^	
Had social support	Yes	49	75.4%	43	66.2%
	No	90	75.6%	80	67.2%
p-value		0.970		0.883	
Type of multiple sclerosis	PRMS	6	100%	6	100%
	RRMS	27	96.4%	26	92.9%
	PPMS	4	80%	5	100%
	FTMS	1	25%	1	25%
	SPMS	5	83.3%	2	33.3%
	Don't know	96	71.1%	83	61.5%
p-value		0.004^a,b^		0.001^a,b^	
Duration of MS	<2 years	37	71.2%	37	71.2%
	>2 years	102	77.3%	86	65.2%
p-value		0.385		0.436	
Treatment situation	Not on treatment	19	76%	19	76%
	Still on treatment	120	75.5%	104	65.4%
p-value		0.954		0.296	
How would you rate your health in general?	Poor	18	85.7%	18	85.7%
	Good	75	85.2%	65	73.9%
	Very good	31	64.6%	29	60.4%
	Excellent	15	55.6%	11	40.7%
p-value		0.002^a^		0.002^a^	
How would you rate your health now compared to last year?	Much better than last year	19	65.5%	16	55.2%
	Somewhat better than last year	32	80%	29	72.5%
	No change	43	69.4%	35	56.5%
	Somewhat worse	26	86.7%	23	76.7%
	Much worse	19	82.6%	20	87%
p-value		0.205		0.028^a^	
Do you think you are depressed now?	Yes	99	94.3%	90	85.7%
	No	40	50.6%	33	41.8%
p-value		0.001^a^		0.001^a^	
Do you think you suffer from an anxiety disorder?	Yes	100	85.5%	94	80.3%
	No	39	58.2%	29	43.3%
p-value		0.001^a^		0.001^a^	

## Discussion

Specific sociodemographic characteristics, such as being female, stresses, parental depression, and specific features, behavior patterns, and dispositions are all contributing causes for depression. These risk factors are linked to biological and genetic causes [16]. As with depression, most anxiety disorders affect women more than men. Anxiety disorders are often accompanied by major depression, alcohol and other substance abuse problems, and personality disorders [17].

Effective therapies for MS can result in fewer depression symptoms, improved psychosocial functioning, and improved quality of life [18]. Therefore, measuring the quality of life in patients with depression is essential when assessing MS therapy outcomes [19].

The high prevalence of psychiatric disorders such as anxiety and depression in the MS population raises a question about the relationship between these conditions. A recent cross-sectional study in Saudi Arabia found that 89.9% of patients with MS suffered from mild to severe depressive symptoms. There was a high risk among unemployed patients (37.39%), and the severity of depression was positively related to education level [3]. Another study done in the United Arab Emirates revealed that 17.6% of patients with MS reported a PHQ-9 score compatible with the diagnosis of depressive disorder, and 20% had a GAD-7 score compatible with anxiety disorder. The study showed no statistical difference in the risk of developed depression or anxiety disorders between the MS and general populations [7]. A study conducted in the United Kingdom using the Hospital Anxiety and Depression Score showed that 54.1% of patients with MS had anxiety, and 46.9% had depression. Patients with SPMS had more severe depression than other MS types [9].

In our study, most participants were female (69%) and aged 35-43 (29.9%) or 26-34 (29.3%). Another study found that women were twice as likely to develop MS than men in general [20], which explains the high female-to-male ratio in our study population. A previous study reported that MS occurred more commonly in patients aged 20-40, which aligns with our study population age distribution [2]. However, another study reported significant variability in the age of diagnosis for patients with MS [20]. A lack of awareness of MS is a widespread issue across Saudi Arabia, as noted in our study and previous studies [21,22]. A minority of participants in our study knew the type of MS they had. In our study, 3.3% of patients with MS had SPMS and 3.3% had PPMS. A previous study found that 12.1% of patients with MS had SPMS, but only 2.6% had PPMS [23]. The likelihood of RRMS turning to SPMS can be reduced with early diagnosis and treatment [23].

We used the PHQ-9 to measure the occurrence and level of depression among our participants. We found that 24.5% of the participants had no or minimal depression, 31% had mild depression, 15.2% had moderate depression, and 12.5% and 16.8% had moderately severe and severe depression, respectively. A study conducted in Saudi Arabia found that 24.8% suffered from mild depression, 23.9% had moderate depression, 22.3% had moderately severe, and 18.9% had severe depression [3]. Biodemographic and environmental differences might explain the variation in the severity of depression and MS types among patients.

Depression peaked in patients aged 18-24 (83.3%), whereas a study in Riyadh found that this same age group had lower percentages of depression ranging from 9.26% to 33.33%, depending on depression severity, but moderate depression was the most common [24]. The participants in our study age 44 or older had more prevalent depression (69.7%) than the findings from a previous cross-sectional study that reported depression in 18.42%-28.95% of patients, depending on depression severity [24].

Anxiety also peaked in the 18-24-year-old group, with a prevalence of 78.6%. Another study found that 23.0% of patients with MS had severe stress, and 44.8% had moderate stress without specifying age groups [2]. One study found that anxiety rates peaked in people older than 60 with up to 50% prevalence for both mild and moderate anxiety [24]. Women with MS were more likely to have depression (83.1%) and anxiety (75.4%) than men, which aligns with two previous studies [2,3]. We found that education level and work status affected depression and anxiety among patients with MS, which supports the findings of a previous study that reported that patients in a good economic state had lower rates of depression and anxiety [2].

In our study, depression was more prevalent among unmarried participants, which contrasts with reports from a study conducted in Iran that reported higher levels of depression in married patients with MS. The effect of marital status may depend on location, community, and culture [2]. Social support did not seem to significantly affect the depression or anxiety rate in our study population.

PRMS had a 100% depression and 100% anxiety rate, indicating that PRMS significantly impacts patients' quality of life. Also, we noted that patients who had lived with MS for more than two years were more likely to be depressed, but patients who were more recently diagnosed with MS had higher rates of anxiety. Treatment had a positive effect on anxiety among patients with MS. This trend points to the need for better educational health systems that will help and encourage patients to develop better coping mechanisms. Given that most study participants reported their health as worsening and many felt depressed or anxious, psychological interventions and screenings must be incorporated into MS treatment protocols.

Limitations and strengths

Our study was limited by the sample size, which restricted data interpretation. Our study was also limited in that we used a self-reported questionnaire to collect data, which may introduce bias and affect the validity of our results. Furthermore, most participants did not know what type of MS disorder they had, which could have affected our results in the correlation between the type of the disease and the anxiety or depression level. Despite the limitations, our study is of public health importance in exploring the quality of care delivered to patients with MS in Saudi Arabia.

## Conclusions

We found a very high rate of depression and anxiety among patients with MS, which highlights the need for periodic screening and examination by psychiatrists to facilitate the early detection and treatment of these comorbidities. Patients with PRMS are at greater risk of developing depression and anxiety than patients with other types of MS. Therefore, patients with PRMS require special attention and additional interventional measures. Patient education programs are essential for mitigating anxiety and depression and addressing quality of life concerns. Further investigations are warranted to determine the impact of depression and anxiety on disease progression and outcomes. Moreover, additional studies are needed to explore the pathological association between MS and psychological disorders.
